# Establishing a Cancer Registry in a Resource-Constrained Region: Process Experience From Ghana

**DOI:** 10.1200/JGO.19.00387

**Published:** 2020-04-17

**Authors:** Joel Yarney, Naomi O. Ohene Oti, Benedict N. L. Calys-Tagoe, Richard K. Gyasi, Isaac Agyeman Duah, Charles Akoto-Aidoo, Valerie McGuire, Julianna C. Hsing, Max Parkin, Yao Tettey, Ann W. Hsing

**Affiliations:** ^1^ Accra Cancer Registry, Accra, Ghana; ^2^ National Centre for Radiotherapy and Nuclear Medicine, Accra, Ghana; ^3^ Department of Community Health, University of Ghana School of Public Health, Accra, Ghana; ^4^ Department of Pathology, University of Ghana, Accra, Ghana; ^5^ Department of Health Policy and Research, Stanford University School of Medicine, Stanford, CA; ^6^ Nuffield Department of Population Health, Oxford University, Oxford, United Kingdom; ^7^ International Agency for Research on Cancer, Lyon, France; ^8^ African Cancer Registry Network, Oxford, United Kingdom; ^9^ Department of Pediatrics, Center of Policy, Outcomes, and Prevention, Stanford University School of Medicine, Stanford, CA

## Abstract

**PURPOSE:**

In a review of cancer incidence across continents (GLOBOCAN 2012), data sources from Ghana were classified as Frequencies, the lowest classification for inclusion, signifying the worst data quality for inclusion in the analysis. Recognizing this deficiency, the establishment of a population-based cancer registry was proposed as part of a broader cancer control plan.

**METHODS:**

The registry was examined under the following headings: policy, data source, and administrative structure; external support and training; and definition of geographic coverage.

**RESULTS:**

The registry was set up based on the Ghana policy document on the strategy for cancer control. The paradigm shift ensured subscription to one data collection software (CanReg 5) in the country. The current approach consists of trained registrars based in the registry who conduct active data abstraction at the departments and units of the hospital and pathologic services. To ensure good governance, an administrative structure was created, including an advisory board, a technical committee, and registry staff. External support for the establishment of the Accra Cancer Registry has come mainly from Stanford University and the African Cancer Registry Network, in collaboration with the University of Ghana. Unlike previous attempts, this registry has a well-defined population made up of nine municipal districts.

**CONCLUSION:**

The Accra Cancer Registry was established as a result of the lessons learned from failed previous attempts and aim to provide a model for setting up other cancer registries in Ghana. It will eventually be the focal point where all the national data can be collated.

## INTRODUCTION

Cancer is the third most common cause of death in developing countries, after cardiovascular and infectious diseases.^1,2^ The magnitude or burden of cancer is small in low- and lower middle–income countries compared with high-income countries, where mortality from cancer is second only to cardiovascular disease. Estimates by the WHO predict an astronomic rise in cancer incidence and mortality in developing countries in the coming years, fueled largely by an increase in life expectancy, lifestyle changes, and success in the management of infectious diseases.^1,2^

Although low-income countries have only 57% of cancer cases, they have 65% of all deaths resulting from cancer.^2,3^ The difference between cancer incidence and mortality is relatively small in low-income countries, and one is more likely to die as a result of the disease in such countries versus in high-income countries, where there is a large difference between incidence and mortality.^4^

Data leading to such conclusions come from cancer registries around the world.^4,5^ The accuracy of estimates and predictions is dependent on the quality of data in these source registries. Not all regions or countries of the world have population-based cancer registries (PBCRs),^5^ including Africa. Cancer registration is a necessity in light of the rising cancer burden in developing countries so as to provide researchers and policymakers with classified information on cancer occurrence to better plan for and control the impact of cancer.^6^ The International Agency for Research on Cancer (IARC) disseminates cancer incidence and mortality data from around the world at regular intervals and therefore relies on data with different levels of accuracy. In the latest publication—GLOBOCAN 2018—the quality of national incidence data is scored from 0 (best) to 10 (worst). Ghana scores 8—meaning that estimates of incidence are based only on local data on the relative frequency of different cancers.^4^ Cancer registration in Africa slowly evolving.^6^ Given the increasing cancer burden and low cancer surveillance, a global initiative for cancer registry development was established by IARC in 2011 to provide expertise and support to cancer registries in regional hubs. As part of this initiative, the African Cancer Registry Network (AFCRN)^8^ was established in 2012 as a regional consortium to support African cancer registries with expertise and to help them surmount challenges and barriers so as to compile good data for decision making and estimations.^9,10^ It also provides regional reports on cancer to support IARC in publishing international cancer estimates.

**CONTEXT**

**Key Objective**
To establish a population-based Cancer Registry in Accra.
**Knowledge Generated**
A model approach for resource-constrained regions/countries to be able to set up population-based cancer registries.
**Relevance**
It is possible for resource-constrained countries to set up a population-based registries that can generate data of acceptable international standards.

In the recent IARC publication “Cancer Incidence in Five Continents, Volume XI,”^11^ approximately 15% of the information came from high-quality cancer registries, with only 1% of the African population covered (ie, the cancer registries meeting the standards set by IARC).^4,10,11^ The coverage percentage for Africa increases to 13% when additional data from PBCRs in sub-Saharan Africa that are part of AFCRN are considered (approximately 25 cancer registries in 20 countries), but these did not meet the inclusion criteria of “Cancer Incidence in Five Continents.”^4,6^ Mortality data estimation is of low or medium quality in Africa, so such data available from PBCRs are used for estimation in some countries, but in those countries without mortality data, estimation is performed by combining cancer incidence with survival probability predicted by country-specific development levels. Incidence rates are derived from local cancer registry reports, mainly covering urban areas. Adjustments are made for underreporting.^4,10,12^

In a review of cancer incidence across continents (GLOBOCAN 2012), the quality of the incidence estimate for Ghana was classified as F, the lowest classification for inclusion, signifying the worst data quality for inclusion in the analysis.^2^ Recognizing this deficiency, the establishment of a PBCR was proposed as part of a broader cancer control plan.^2^ Several unsuccessful attempts have been made in the past to establish a registry; our latest attempt has been bolstered by grant and technical support from Stanford University beginning on September 1, 2016.

The objective of this article is to document challenges and successes before and after the latest attempt at establishing a PBCR in Ghana.

## METHODS

The registry was examined under the following headings: policy, data source, and administrative structure; external support and training; and definition of geographic coverage.

## RESULTS

### Policy

Ghana has a policy document on its strategy for cancer control. This policy document outlines how a PBCR should be set up and how it fits into the broader context of cancer control.^13^ The registry was therefore positioned in the new attempt in the broader context of a country cancer control program. In contrast, previous attempts had involved standalone programs, with no particular plan to merge data across the country. This paradigm shift ensured subscription to one data collection software (CanReg 5) in the country.

### Data Source

Before the recent attempt, data were obtained from individual departments in the Korle-Bu Teaching Hospital, the largest and top referral hospital in the country, with a bed capacity of 2,000, receiving patients from across the country.

The following departments in the hospital recorded information on cancer statistics in various formats of their choice: Oncology, Pathology, Child Health, Surgery, Gynecology, and Hematology. Information on specific cancers could also be obtained through medical record reviews in various departments. These data sources have resulted in several publications,^14-26^ however, this disparate approach fails to capture cancer incidence and mortality data with a good degree of certainty.

In 2001, an attempt was made at establishing a PBCR with the assistance of IARC and championed by the Non-Communicable Disease Division (NCD) of the Ministry of Health in Ghana. An office was created outside of the hospital and equipped with computers and software provided by IARC.

Existing staff of NCD, who received very little on-site training, were engaged for data abstraction and entry. Information was obtained mainly from the Oncology Department of the Korle-Bu Teaching Hospital. The site was supervised by a pathologist and the director of NCD. This attempt ended as visits by registrars from NCD to the Oncology Department for data slowly tapered off. The data collected at the time was biased toward cases that required radiotherapy and chemotherapy. Surgical, hematologic, and childhood cancers were underreported.

In 2011, another attempt was made at reviving the registry; this time, the services of public health nurses stationed in various departments within the hospital were used to collect cancer-related information from patient medical records and physician interviews. Once again, these officers failed to demonstrate the required dedication to the strict collection of information. The scanty information that was recorded was added to data from the Oncology Department, resulting in an apparent bias toward patients requiring radiotherapy and chemotherapy.^24^

The current approach consists of trained registrars based in the registry who actively visit all the departments and units in the hospital to abstract data from patient medical records on a schedule.

A second data source is the death registry at the Korle-Bu Teaching Hospital Pathology Department and pathology reports from the recognized pathology laboratories within our catchment area. We have taken care to include the accession numbers of reports in the abstracted data to allow ease of linkage between the registry data and pathologic samples. The Data Supplement shows a sample of the data abstraction form used to collect data, and Table 1 summarizes data collected from various departments in the hospital before and after recent changes. It is obvious that the number of data sources and the number of abstractions have improved considerably. The basis for diagnosis is summarized in Table 2.

**TABLE 1:**
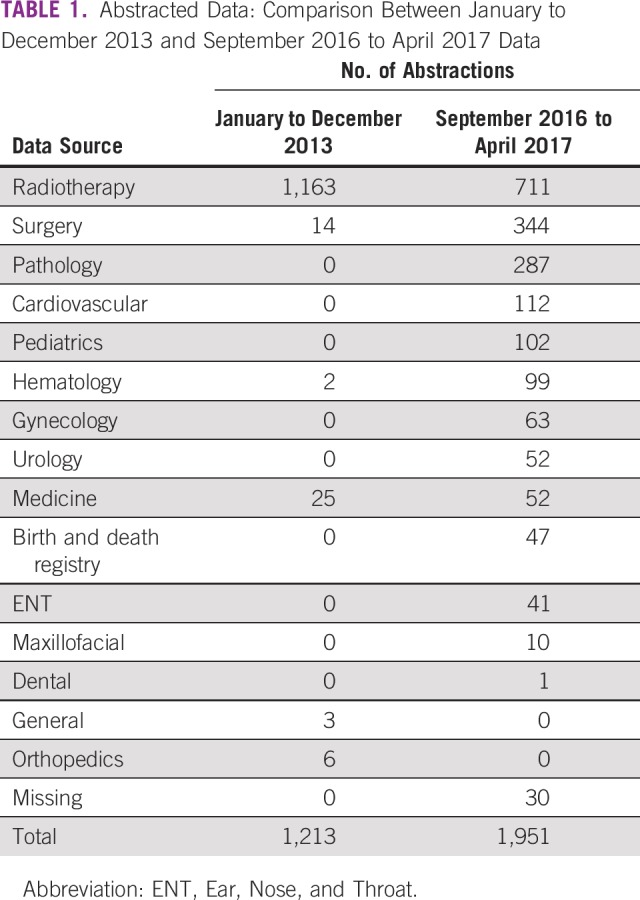
Abstracted Data: Comparison Between January to December 2013 and September 2016 to April 2017 Data

**TABLE 2:**
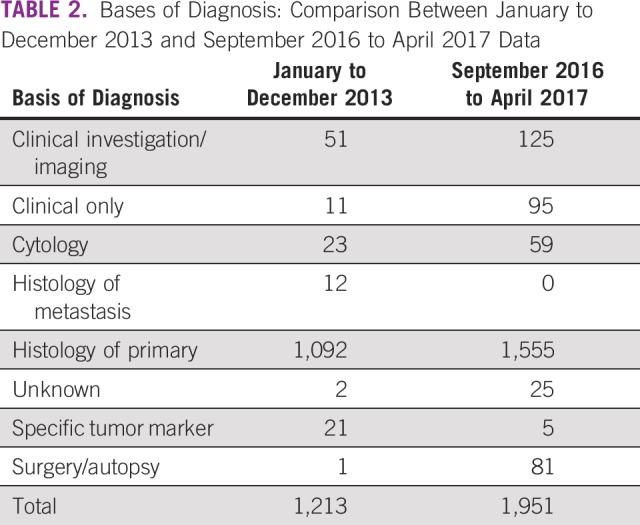
Bases of Diagnosis: Comparison Between January to December 2013 and September 2016 to April 2017 Data

### Administrative Structure

**FIG 1:**
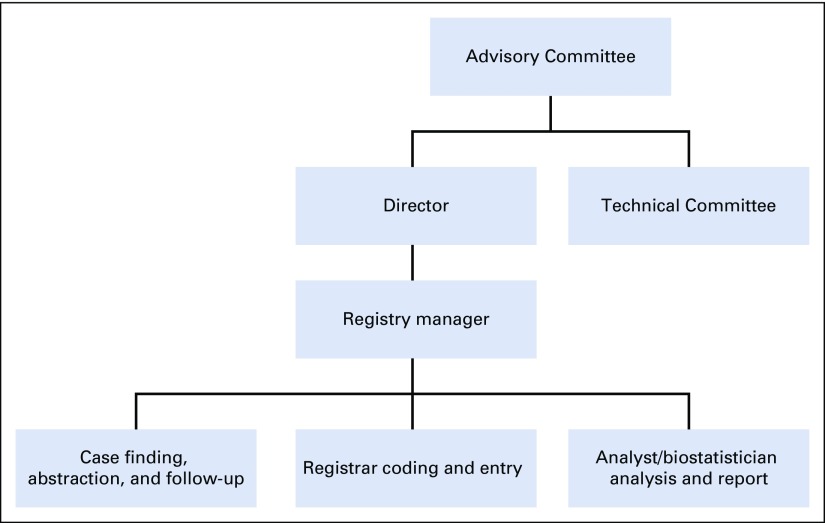
Administrative structure of the Accra Cancer Registry.

NCD was responsible for the day-to-day administration of the registry in the initial attempt. To ensure good governance, an administrative structure was created, shown in Figure 1. The membership of the Advisory Board comprises the following: heads of the Departments of Pathology, Oncology, Surgery, Child Health, Gynecology, Internal Medicine, and Public Health; representatives from the birth and death registry, statistical service, private laboratories, and private hospitals; an epidemiologist; the director of the registry; the program manager of NCD; the registry manager; and a senior registrar who as the secretary.

The advisory board is responsible for determining the strategic direction of the registry, staff recruitment and training, review of registry reports, and fund raising. The registry manager is responsible for the day-to-day administration of the registry and data validity and security; he or she reports to the director of the registry. The director provides supervision of the registry staff through the manager. He or she forms the link between the Advisory Board and Technical Committee and the registry staff and is therefore a member of these committees. The registrars are responsible for case finding, abstraction, coding, entry, and collation. The Technical Committee comprises a pathologist, an oncologist, an epidemiologist, and the registry manager; they are also members of the Advisory Board.

### External Support and Training

External support for the establishment of the Accra Cancer Registry has come mainly from Stanford University and AFCRN in collaboration with the University of Ghana School of Medicine and Dentistry.

An initial assessment of installed capacity to establish the registry was performed in 2001, but it failed to translate into action. Subsequently, the recent attempt began with two visits by consultants from AFCRN followed by recommendations for a workshop.

The workshop provided training on the basic background of cancer epidemiology and registration, including the terminology, structure, and function of a cancer registry. Other topics considered included basic methodology related to data collection and registration, CanReg 5 software for data recording and tabulation, evaluation and assessment of cancer registration and data quality, and a brief on the International Classification of Diseases for Oncology.

Stanford University, through its grant to the University of Ghana School of Medicine and Dentistry, also provided expertise in the formulation of a data release instrument, including confidentiality and a data-sharing policy and agreement. It helped in creating a tumor registry and the platform for linkage between the cancer registry and the tumor registry.

### Coverage Area

**FIG 2:**
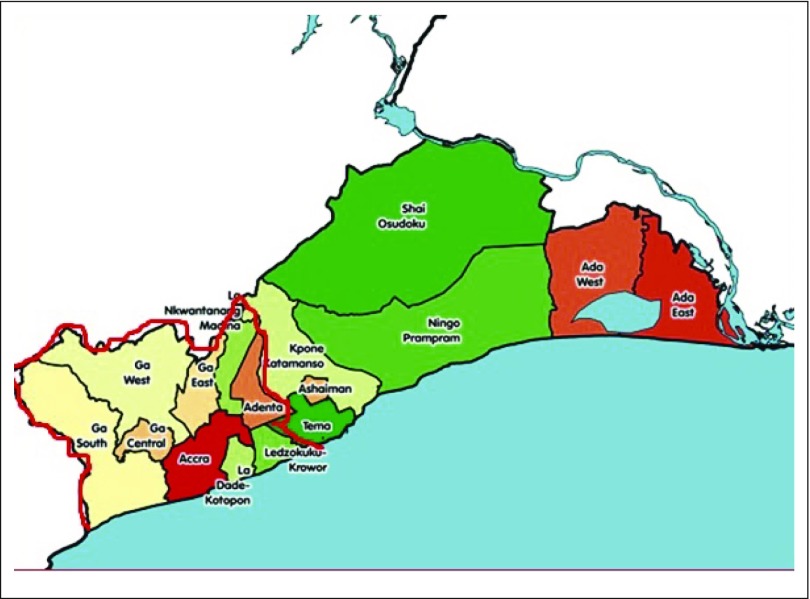
Defined population area of the Accra Cancer Registry in the greater Accra region (2010 Census, Ghana), outlined in red, including Accra, Adentan, Ga South, Ga East, Ga West, Ga Central, La-Dadekotokpon, La-Nkwantanang, and Ledzokukukrowor municipal assemblies.

Unlike previous attempts, in which any patient with cancer who visited the Korle Bu Teaching Hospital was considered a case, resulting in the calculation of incidence and mortality rates by extrapolation from hospital data with respect to total number of patients visiting the hospital as the denominator,^24^ our recent attempt requires careful recording of place or residence, so that patients can be related to a defined geographic area for the calculation of incidence and mortality rates. This created an opportunity to use age-specific population data in the defined region to derive incidence rates. It became necessary to rename the hospital registry the Accra Cancer Registry to reflect the jurisdiction of operation and the population-based nature of the registry. It was determined to include major hospitals and pathology laboratories in the jurisdiction likely to manage patients with cancer. In Ghana, there are 267 hospitals, with approximately 76 in the greater Accra region,^27^ which includes our defined area covering approximately 30% of the total hospitals in the greater Accra region. The area of operation is outlined in Figure 2 with a red border together with the districts to be covered. In particular, the Ghana Sweden Medical Centre, which is a cancer center, was carefully included in the area to be covered. The total population of the coverage area is 2,748,401.^28^ There are some challenges that may hinder the process of registration and data abstraction, including a lack of organized medical records, unrecorded cancer cases at health facilities because of nonattendance, and a poor address system making it difficult to conduct follow-up studies.

The financial problem of cancer registration may far outweigh the structural and technical hindrances. This is reflected by the difficulty in obtaining local funding for registry activities.

## DISCUSSION

The “Global Action Plan for Prevention and Control of Noncommunicable Disease 2013-2020”^29^ document recommends a number of policy actions under objective 6 with regard to monitoring trends and determinants of noncommunicable disease and evaluating progress in their prevention and control. It recommends the development of disease registries and mentions cancer in particular. The importance of a cancer registry that produces data of good quality that can be relied upon for the monitoring of trends and evaluation of progress in the prevention and control of cancer cannot be overemphasized.

The capacity of Ghana with respect to cancer registration is summarized by the report on data quality from Ghana in the IARC publications of GLOBOCAN 2012 and 2018. To improve upon this observation, Ghana has undertaken the establishment of at least one registry each in the southern, middle, and northern zones of the country. The Accra Cancer Registry is located in the southern zone. The prediction of an astronomic increase in the burden of cancer, particularly in low-income countries, demands that a concerted effort be made by such countries to prevent and control the disease.

To be successful, support from government is important; in addition to procedural flaws in previous attempts to establish a cancer registry, the lack of support from official government channels has hampered the smooth establishment of a registry. Specifically, most of the effort made in establishing the registry has come from individual interest and effort. The registry has yet to be recognized as an entity and therefore be provided with the requisite financial or administrative support.

A proper administrative setup is crucial for the success of a cancer registry. It should have dedicated staff for case finding; otherwise, many cases may go unidentified. The Advisory Board, Technical Committee, director, and manager together constitute an important set up to ensure the smooth running of the registry. Data sources should include hospital as well as pathology services, death registry, and autopsy. It would be expedient to mandate pathology laboratories to provide notification of cancer diagnoses to the registry.

When fully established, our registry will provide a good platform for research into the determinants of cancer in Ghana to contribute to the global picture. It is important to position the registry in the broad context of a national cancer control and prevention strategy to facilitate the necessary linkages that are required to ensure a holistic approach. Patient records of risk factors and other sociodemographic considerations are useful in determining patterns of occurrence. An efficient system for cause-of-death registration is important to determine the burden of cancer as a percentage of all-cause mortality as well as to determine progress in prevention and treatment strategies. Death registration in Ghana stands at 20%^30^and needs to be improved to feed into the quality of registry data. The data on risk factors may be useful in determining causation and monitoring trends.

Training and technical support are indispensable in the formative stages of a registry. In this regard, AFCRN and Stanford University have played a crucial role.

The choice of districts to include in the registry includes a mixture of rural and urban settings, because these may differ in their patterns of cancer as well as sociodemographic characteristics, thus enabling a complete picture to be determined for the geographic area.

In conclusion, the Accra Cancer Registry, which has been established as a result of the lessons learned from failed previous attempts and through a grant and technical support from Stanford University, is a PBCR with the added advantage of a tumor registry in a principal national referral hospital. It should be a model for establishing other cancer registries in Ghana and will eventually be the focal point where all the national data can be collated.
